# The New York Head—A precise standardized volume conductor model for EEG source localization and tES targeting

**DOI:** 10.1016/j.neuroimage.2015.12.019

**Published:** 2015-12-17

**Authors:** Yu Huang, Lucas C. Parra, Stefan Haufe

**Affiliations:** a Department of Biomedical Engineering, City College of the City University of New York, New York, NY 10031, USA; b Laboratory for Intelligent Imaging and Neural Computing, Columbia University, New York, NY 10027, USA; c Machine Learning Department, Technische Universität Berlin, 10587, Berlin, Germany

**Keywords:** ICBM-NY, Volume conductor, Head model, Forward model, Lead field, Finite element model (FEM), Electroencephalography (EEG), Inverse source imaging, Transcranial electric current stimulation (tES), Targeting, Boundary element model (BEM), Spherical harmonics expansion (SHE)

## Abstract

In source localization of electroencephalograpic (EEG) signals, as well as in targeted transcranial electric current stimulation (tES), a volume conductor model is required to describe the flow of electric currents in the head. Boundary element models (BEM) can be readily computed to represent major tissue compartments, but cannot encode detailed anatomical information within compartments. Finite element models (FEM) can capture more tissue types and intricate anatomical structures, but with the higher precision also comes the need for semiautomated segmentation, and a higher computational cost. In either case, adjusting to the individual human anatomy requires costly magnetic resonance imaging (MRI), and thus head modeling is often based on the anatomy of an ‘arbitrary’ individual (e.g. Colin27). Additionally, existing reference models for the human head often do not include the cerebrospinal fluid (CSF), and their field of view excludes portions of the head and neck—two factors that demonstrably affect current-flow patterns. Here we present a highly detailed FEM, which we call ICBM-NY, or “New York Head”. It is based on the ICBM152 anatomical template (a non-linear average of the MRI of 152 adult human brains) defined in MNI coordinates, for which we extended the field of view to the neck and performed a detailed segmentation of six tissue types (scalp, skull, CSF, gray matter, white matter, air cavities) at 0.5 mm ^3^ resolution. The model was solved for 231 electrode locations. To evaluate its performance, additional FEMs and BEMs were constructed for four individual subjects. Each of the four individual FEMs (regarded as the ‘ground truth’) is compared to its BEM counterpart, the ICBM-NY, a BEM of the ICBM anatomy, an ‘individualized’ BEM of the ICBM anatomy warped to the individual head surface, and FEMs of the other individuals. Performance is measured in terms of EEG source localization and tES targeting errors. Results show that the ICBM-NY outperforms FEMs of mismatched individual anatomies as well as the BEM of the ICBM anatomy according to both criteria. We therefore propose the New York Head as a new standard head model to be used in future EEG and tES studies whenever an individual MRI is not available. We release all model data online at neuralengr.com/nyhead/ to facilitate broad adoption.

## Introduction

Today, a multitude of tools are available to non-invasively ‘read and write the brain.’ Brain imaging technologies such as electroencephalography (EEG) allow one to track the activity of neuronal populations with millisecond precision. Conversely, transcranial electric stimulation (tES) induces changes in neuronal firing patterns by injecting electric currents into the scalp. What is common to these technologies is that they rely on a volume conductor model of the human head to establish the connection between structures in the brain and electrodes located on the scalp. The ‘lead field’ or ‘forward model’ used for EEG inverse modeling relates a current source in the brain to the electric potentials measured on the scalp (Sarvas, 1987; Mosher et al., 1999; Baillet et al., 2001; Vatta et al., 2010; Akalin Acar and Makeig, 2013; Vorwerk et al., 2014). What is called ‘forward model’ in tES captures the electric field generated in the brain when applying current to scalp electrodes (Wagner et al., 2007; Datta et al., 2009, 2012; Mendonca et al., 2011; Dmochowski et al., 2013). According to the reciprocity theorem, the two forward models are identical (Rush and Driscoll, 1969), so that the terms ‘forward model’ and ‘lead field,’ as well as ‘volume conductor model’ and ‘head model,’ are interchangeable. The accuracy of such a model determines the precision of both source localization in EEG and targeting of specific brain structures using tES.

Volume conductor models are commonly formulated as boundary element models (BEM) or finite element models (FEM). The classic three-shell BEM is currently the predominant approach in EEG source imaging (Mosher et al., 1999) because of its computational efficiency, and because it can be readily constructed from structural magnetic resonance images (MRI) using several freely available software packages such as LORETA (Pascual-Marqui et al., 1994; Fuchs et al., 2002), BrainVISA (Rivière et al., 2003; Geffroy et al., 2011), EEGLAB-NFT (Acar and Makeig, 2010), OpenMEEG (Gramfort et al., 2010), MNE (Gramfort et al., 2014), Brainstorm (Tadel et al., 2011), and FieldTrip (Oostenveld et al., 2011). In the BEM, the major tissues (brain, skull, scalp) are represented by tissue boundaries derived from the individual’s anatomy. However, BEMs are limited by the constraint that boundaries must entirely enclose each other forming ‘shells’ and that they must be reasonably smooth. Additionally, the cerebrospinal fluid (CSF) is often not included, because most current automated segmentation tools do not resolve the thin CSF layer. All of these limit the anatomical realism and accuracy of BE current-flow modeling (Vorwerk et al., 2014).

Most tES research use FEMs instead to encode finer anatomical details more accurately at the resolution of the MRI. This includes the gyri/sulci of the cortex, the thin layer of CSF, and the small but delicate structures of the skull (Datta et al., 2009, 2010, 2012; Mendonca et al., 2011).

As head anatomies vary greatly across the population, individual structural information from MRI is required to build precise volume conductor models. However, the acquisition of individual MRI is not always possible and generally comes at a high cost. Further complicating matters, detailed finite element modeling requires manual intervention in the segmentation process (Datta et al., 2009, 2012). Despite the recent efforts to automate the segmentation (Huang et al., 2013; Huang and Parra, 2015), and the FEM processing pipeline (Wolters et al., 2007; Windhoff et al., 2011; Dannhauer et al., 2012), there is still no fully automated tool available for individualized FE modeling. Therefore, it is a common practice in the tES community to use a detailed FEM built from an ‘arbitrary’ individual as a reference model (Villamar et al., 2013; Truong et al., 2014; Richardson et al., 2014; Jones et al., 2015).

The most commonly used individual model is Colin27 (Holmes et al., 1998), an average of 27 MRI scans of Colin J. Holmes. A BEM of the Colin27 head is included in many neuroimaging software packages, such as LORETA (Pascual-Marqui et al., 1994), EEGLAB-NFT (Acar and Makeig, 2010), Brainstorm (Tadel et al., 2011), and FieldTrip (Oostenveld et al., 2011). An FEM of Colin27 has also been used previously for tES. However, respective studies did not differentiate the CSF from the brain (Park et al., 2011; Jung et al., 2013) or used a limited field of view (FOV) (Salvador et al., 2010). The main problem with such reference models, however, is the obvious bias introduced by using an arbitrary individual head, which is present even for templates warped to a standard space such as the MNI space defined by the Montreal Neurological Institute.

Here we reason that, while in the near future it may remain infeasible to compute highly detailed FEMs in individual anatomies at the scale of larger studies, an improvement may already be achieved by replacing arbitrary templates with an unbiased population average. Currently, the best available average over a population of individuals is the so-called ICBM152 head of the International Consortium for Brain Mapping (Mazziotta et al., 1995, 2001a, 2001b; Grabner et al., 2006; Fonov et al., 2009, 2011), which, thanks to advances in non-linear image registration, has achieved a level of detail comparable to that of an individual head.

We built an FEM based on the ICBM152 head to be used for EEG source imaging as well as tES targeting. Specifically, we combined the highly detailed brain image of the ‘non-linear’ ICBM152 v2009b template (0.5 mm^3^ resolution, (Fonov et al., 2009, 2011)) with the high-quality image of the non-brain area of the ICBM152 v6 template (1 mm^3^ resolution, (Grabner et al., 2006)). The FOV of the combined model was extended down to the neck using an additional average head of 26 subjects provided by Chris Rorden (Huang et al., 2013). This composite model, which we term ICBM-NY,^1^ alias the ‘New York Head,’ includes scalp, skull, CSF, gray matter, white matter, and air cavities. To circumvent slow-processing times of detailed FEMs, the lead fields were precomputed and stored for 231 electrodes on the scalp following the international 10–05 system. Performance of this ICBM-NY head was evaluated by comparing it to FEMs of similarly detailed segmentations obtained from four individuals, which are used alternately as ‘ground truth,’ or ‘reference,’ heads. Additional comparisons were performed with computationally efficient BEM and spherical harmonics expansions (SHE, (Nolte and Dassios, 2005; Marzetti et al., 2008; Haufe et al., 2008, 2011)) models of the reference anatomy, a BEM of the ICBM152 anatomy, as well as ‘individualized’ BEMs that are adjusted to the individual outer shape of the head (Leahy et al., 1998; Darvas et al., 2006; Acar and Makeig, 2010), which is more readily available via 3D digitization hardware than individual MRIs. Performance metrics include deviations of the lead fields from the ground truth, EEG localization accuracy, as well as tES targeting accuracy.

## Methods

### MRI acquisition and preprocessing

The McConnell Brain Imaging Centre of the Montreal Neurological Institute (MNI, Montreal, Canada) provides three templates of human heads^2^: MNI-305, Colin27, and ICBM152. MNI-305 (Evans et al., 1993; Collins et al., 1994) is a linear average of the T1-weighted structural MRIs of 305 human heads. This average blurs the anatomical details needed for realistic current-flow modeling. Colin27 (Holmes et al., 1998; Aubert-Broche et al., 2006) is an average of 27 MRI scans of a single individual and may thus provide biased results. The ICBM152 template is an unbiased non-linear average of MRIs of 152 adult human subjects, of which several versions exist. The older version, ICBM152 v6, better preserves detail of the skull and scalp anatomy (Mazziotta et al., 2001a; Grabner et al., 2006). The newer version, ICBM152 v2009b, better preserves anatomical details of the brain (Fonov et al., 2009, 2011). Both come in a symmetric and a regular version. As outlined in more detail in Segmentation and electrode placement section, the symmetric versions of the ICBM152 v2009 and the ICBM152 v6 in combination with another average of 26 heads provide the anatomical basis for our model, which we call ICBM-NY, alias, the ‘New York Head.’

We also acquired MRI (1 mm^3^ isotropic resolution, T1-weighted) of four healthy individuals (denoted INDV1–4, all Caucasian male, age range 27–45) at a magnetic field of 3 T. INDV1 was scanned in a Siemens Trio scanner (Erlangen, Germany) using a gradient echo (GRE) sequence with TE = 4.2 ms, TR = 2250 ms, 256 × 256 matrix scan with 176 sagittal slices. INDV2 was also scanned in a Siemens Trio scanner using a GRE sequence with TE = 2.3 ms, TR = 1900 ms, 280 × 320 matrix scan with 208 sagittal slices. INDV3 was scanned in a General Electric Signa Excite HD scanner (Fairfield, CT) using a GRE sequence with TE = 2.2 ms, TR = 7.3 ms, 256 × 256 matrix scan with 252 axial slices. INDV4 was scanned in a Siemens Trio scanner using a magnetization prepared rapid acquisition gradient echo (MPRAGE) sequence with TE = 2.98 ms, TR = 2300 ms, 240 × 256 matrix scan with 160 sagittal slices.

All four individual MRIs were registered to the ICBM152 v6 head template using the ‘Coregister’ function (Collignon et al., 1995) provided by the Statistical Parametric Mapping (SPM8) package (Wellcome Trust Centre for Neuroimaging, London, UK) in Matlab (The Mathworks, Natick, MA). The registration yielded a 6-parameter affine transform consisting of a rotation and translation, but no scaling or shearing. This transform, *M*_1_, defines a native reference-space with the origin located at the anterior commissure for each subject. All lead fields and other data reported in the following are expressed in these native-space coordinates.

In addition to the MRI-to-native transform, a 12-parameter affine transform (*M*_2_) from the individual native space to the MNI reference-space defined by the MNI-305 template (Evans et al., 1993; Collins et al., 1994) was calculated for each individual using the ‘Normalise’ function (Friston et al., 1995) in SPM8. These transforms were used later to match cortical locations in different anatomies (see Mapping between cortical locations of different anatomies section).

Notice that none of the above-mentioned transforms was applied to the actual MRI data. Both were only stored for later usage. Moreover, notice that the native space of the ICBM152 head is by construction aligned with the MNI space.

### Segmentation and electrode placement

The two versions of the ICBM152 (v6 and v2009b), as well as the four individual heads (INDV1–4) were segmented using a probabilistic segmentation routine (New Segment, an extension of Unified Segmentation, (Ashburner and Friston, 2005)) in SPM8. For the anatomical prior probability, we used a tissue probability map (TPM) developed by Chris Rorden (CR-TPM, (Huang et al., 2013)). This resulted in a segmentation of six tissue types: gray matter (GM), white matter (WM), CSF, skull, scalp, and air cavities. A custom Matlab script was used to correct for segmentation errors made by SPM, such as rough tissue surfaces, discontinuities in CSF, and skull layers, and disconnected regions (Huang et al., 2013). The remaining errors in continuity and anatomical details were manually corrected in ScanIP 4.2 (Simpleware Ltd., Exeter, UK).

**Fig. 1:**
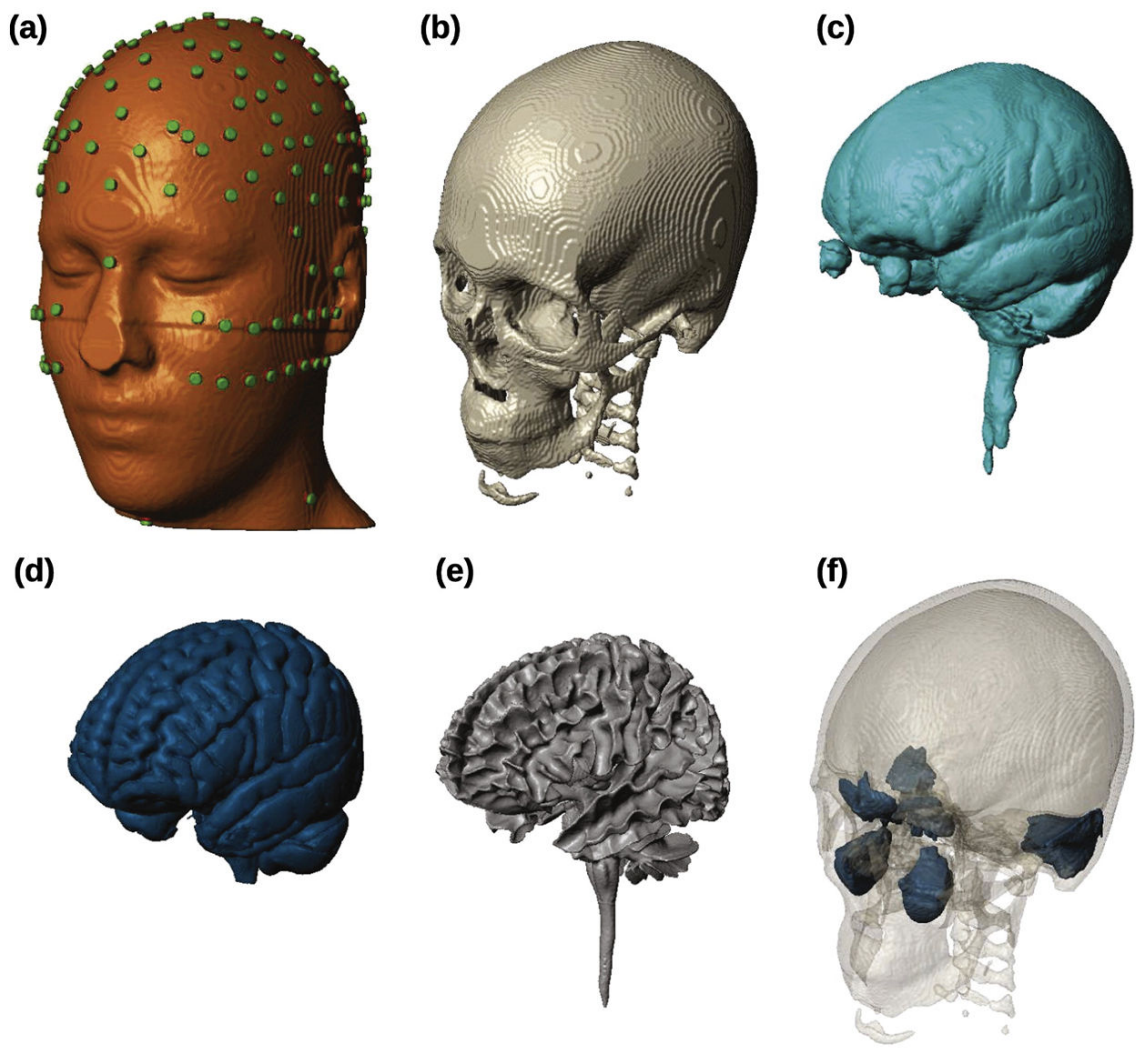
Segmentation of the ICBM-NY head into six different tissue types. From (a) to (f): scalp (with 231 electrodes placed), skull, cerebrospinal fluid, gray matter, white matter, air cavities. Note that the disc electrodes and underlying gel in (a) are not physically modeled. Instead, they are represented by a single tetrahedral mesh-element on the scalp surface.

Since the ICBM152 v2009b is characterized by a higher resolution and better image quality in the brain, but poorer quality in the non-brain region compared to the ICBM152 v6, the non-brain tissues (CSF, skull, scalp, air) obtained from ICBM152 v6 were registered to the MRI space of ICBM152 v2009 using SPM’s Coregister routine, and resliced. This process performs generally well except that some of the voxels in the resliced CSF overlap with brain (mainly GM) voxels. The overlapping parts of the CSF were removed from the brain by Boolean subtraction, resulting in discontinuities of the CSF surface. To correct for this, the CSF was combined with the brain, dilated by a spherical structural element of 1 mm diameter, and then subtracted from the brain. Residual overlap of CSF and skull was subtracted from the CSF, and resulting discontinuities on the skull were manually corrected by subtracting voxels from the scalp. After these operations, a combined ICBM152 head with 0.5 mm^3^ resolution and abundant anatomical details in both brain and non-brain tissues was obtained. The FOV of this combined image, however, only covers the brain area. tES modeling work has demonstrated the need to include the entire head down to the neck for realistic current flow, in particular in deep-brain areas and the brainstem (Huang et al., 2013). To this end, the CR-TPM, which has an FOV covering the whole head, was registered to the voxel space of the ICBM152 v2009 template, resliced, and fused inferiorly to the combined ICBM152 head. Thus, we fused the brain (GM, WM) obtained from ICBM152 v2009b with the non-brain tissues obtained from ICBM152 v6 and the lower head obtained from CR-TPM into a new, high-resolution (0.5 mm^3^), whole-head model referred to as the ICBM-NY (New York) head. 3D renderings of the tissue compartments of the ICBM-NY are shown in Fig. 1.

For all heads, electrodes were placed on the scalp surface automatically using a custom Matlab script described in (Huang et al., 2013). Specifically, we used a subset of the 165 electrode locations defined in the 10–05 system (Oostenveld and Praamstra, 2001).

In addition, two rows of electrodes below the ears and four additional electrodes around the neck were placed to allow for targeting of deeper cortical areas, and for the use of distant reference electrodes in tES. To avoid complications when automatically placing electrodes near or behind the ear-lobes, the electrodes TP9 and TP10 were omitted. A total of 231 electrodes were placed for each head (see Fig. 1).

Note that the electrode modeling differs here from what is described in (Huang et al., 2013). We did not physically model the electrodes and the underlying gel, because, due to the dense electrode montage considered, the proximity of the electrodes on the scalp surface would artificially increase surface conductance. Instead, each ‘electrode’ is represented as a small triangular area corresponding to the surface of the closest tetrahedral mesh-element (see below).

### Finite element modeling

A FEM with adaptive tetrahedral element sizes was generated for each head using ScanIP (+ScanFE Module, ScanFE-Free algorithm). Laplace’s equation (−∇·(*σ***E**)=0) was then solved (Griffiths, 1999) in Abaqus 6.11 (SIMULIA, Providence, RI) for the electric field distribution **E** in the head. Each tissue type was assigned a conductivity *σ* as in Huang et al. (2013). The boundary conditions were set to: insulated on the scalp surface, grounded on electrode location Iz, and 1 A/m^2^ of inward current density on each of the other electrode locations. Thus, for each head, we obtained 230 solutions for electric field distribution representing the ‘forward model’ or ‘lead field.’ For subsequent analyses, gray matter voxels were extracted. The lead fields evaluated at these voxels were calibrated to correspond to 1 mA current injections from the scalp surface, whereas the corresponding MRI voxel coordinates were converted into the native coordinate system of each head using the individual transform matrix *M*_1_ (Fig. 3).

Note that by including CSF and air cavities and by distinguishing between gray and white matter, we here closely follow the guidelines for precise electrical modeling of the head formulated by Vorwerk et al. (2014), who identified these factors as being more important than the distinction of skull spongiosa and compacta, as well as the modeling of white matter anisotropy.

### Boundary element and spherical harmonics modeling

**Fig. 2:**
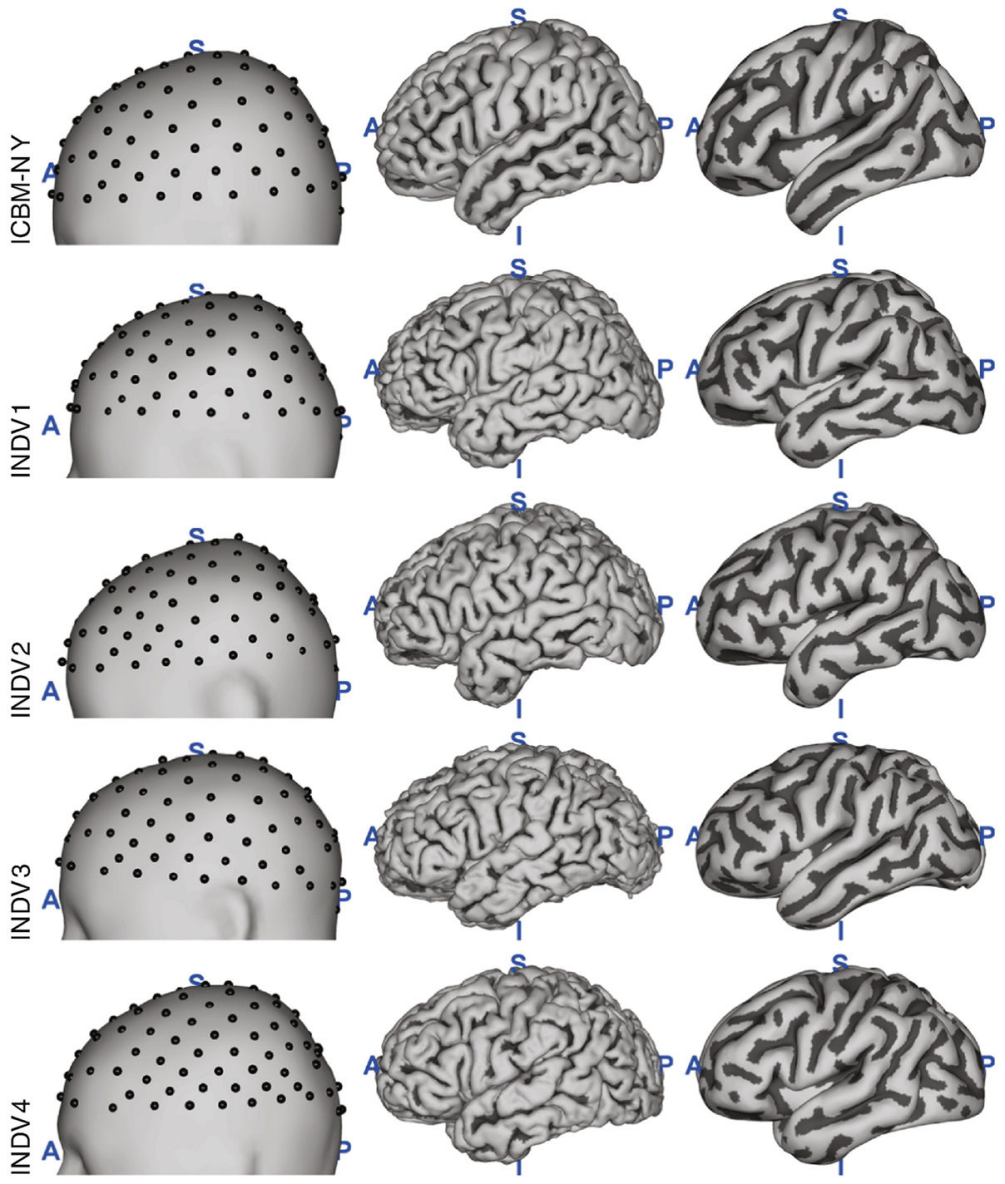
The ICBM-NY anatomy as compared to four individual heads (INDV1–4). Left: head (outer shell of a BEM model) surface with the subset of the 108 electrodes used for the quantitative evaluation. Center: cortical surface. Right: smoothed cortical surface used for plotting. Cortical sulci are marked in dark color.

For the purpose of comparison, we generated BEMs using conventional procedures as follows. Using the ‘Morphologist’ pipeline of BrainVISA (http://brainvisa.info/), high-resolution meshes of the cortical surface were obtained (with about 75,000 nodes) for all four individual heads, as well as the ICBM152 v2009 head from their T1-weighted MR images. Fig. 2 shows the extracted cortical surfaces. Note that the smoothed surfaces shown in the right panel of the figure are solely used for plotting. Surfaces meshes of the brain, skull, and scalp compartments comprising 1922 nodes each were extracted using the Brainstorm package (Tadel et al., 2011). Within this 3-shell geometry, the EEG forward problem was solved using BEM as implemented by the OpenMEEG package (Gramfort et al., 2010), as well as using spherical harmonics expansions (SHE) of the electric lead fields (Nolte and Dassios, 2005). The electrical conductivities used for the brain, skull, and scalp compartments were *σ*_1_ =0.33 S/m, *σ*_2_ =0.041 S/m, and *σ*_3_ =0.33 S/m, respectively.

Note that we used the ‘regular’ ICBM152 head for BEM and SHE modeling to demonstrate what results would be obtained using existing freely available toolboxes. However, since these models rely on a three-shell geometry, key features of the ICBM-NY such as an extended FOV, inclusion of CSF, and a highly detailed skull are largely ignored. Specifically, the outer shells generated by Brainstorm are cut off a few centimeters below the brain. Moreover, a constant skull thickness of 4 mm is assumed, and the CSF is omitted. We would therefore expect similar BEM/SHE results for the ICBM152 and ICBM-NY anatomies.

### Generation of individualized warped ICBM templates

In addition to the ICBM152 and the INDV1–4 heads, four individualized versions of the ICBM152 template were constructed by warping it to match the individual shape of the scalp. To this end, the ICBM152 head surface was morphed to fit the electrodes locations on each of the four individual heads INDV1–4 (Leahy et al., 1998; Tadel et al., 2011). The warping was carried out in Brainstorm. Note that building such models is possible in practice using 3D digitization hardware without requiring any individual structural MRI data. The estimated warping transformations were subsequently applied to all precomputed surfaces of the ICBM152 head. Lead fields were computed in these warped anatomies using BEM (OpenMEEG toolbox), giving rise to four ‘individualized’ (as opposed to ‘individual,’ which refers to the use of individual structural MR images) head models.

### Quantitative comparison of head models

We quantitatively evaluated how well the proposed ICBM-NY head model approximates the current flow in the individual heads INDV1–4 and compared this to other commonly used head models. For this study, the FEM calculated in each individual anatomy was regarded as the ‘ground truth’ for that individual and will be referred to as the ‘reference head model’ (REF FEM). Head models differing from REF FEM are called approximate and can arise for two reasons: 1) an incongruent anatomical basis (as is the case if we use a different individual for comparison) and 2) an electrical model different from FEM (e.g., a BEM, which can only approximate the more detailed FEM even it is applied to the reference anatomy).

Besides the ICBM-NY, we evaluate the following head models against the ground truth provided by REF FEM: a BEM and a SHE electrical model of the reference anatomy (denoted as REF BEM and REF SHE, respectively), FEMs of three other individuals’ anatomies (summarized under the term INCG FEM), a BEM of the ICBM152 anatomy (ICBM BEM), and an ‘individualized’ BEM of the ICBM152 anatomy (denoted as WARP BEM). All lead fields were re-referenced to the common average of the selected channels. A subset of 108 electrode locations was selected for the lead field comparisons, and EEG source localization study described below. The distribution of these electrodes across the scalp is shown in Fig. 2 for all heads. For the tES targeting study described in Assessment of tES targeting accuracy section, the full set of 231 electrodes was used.

### Mapping between cortical locations of different anatomies

**Fig. 3:**
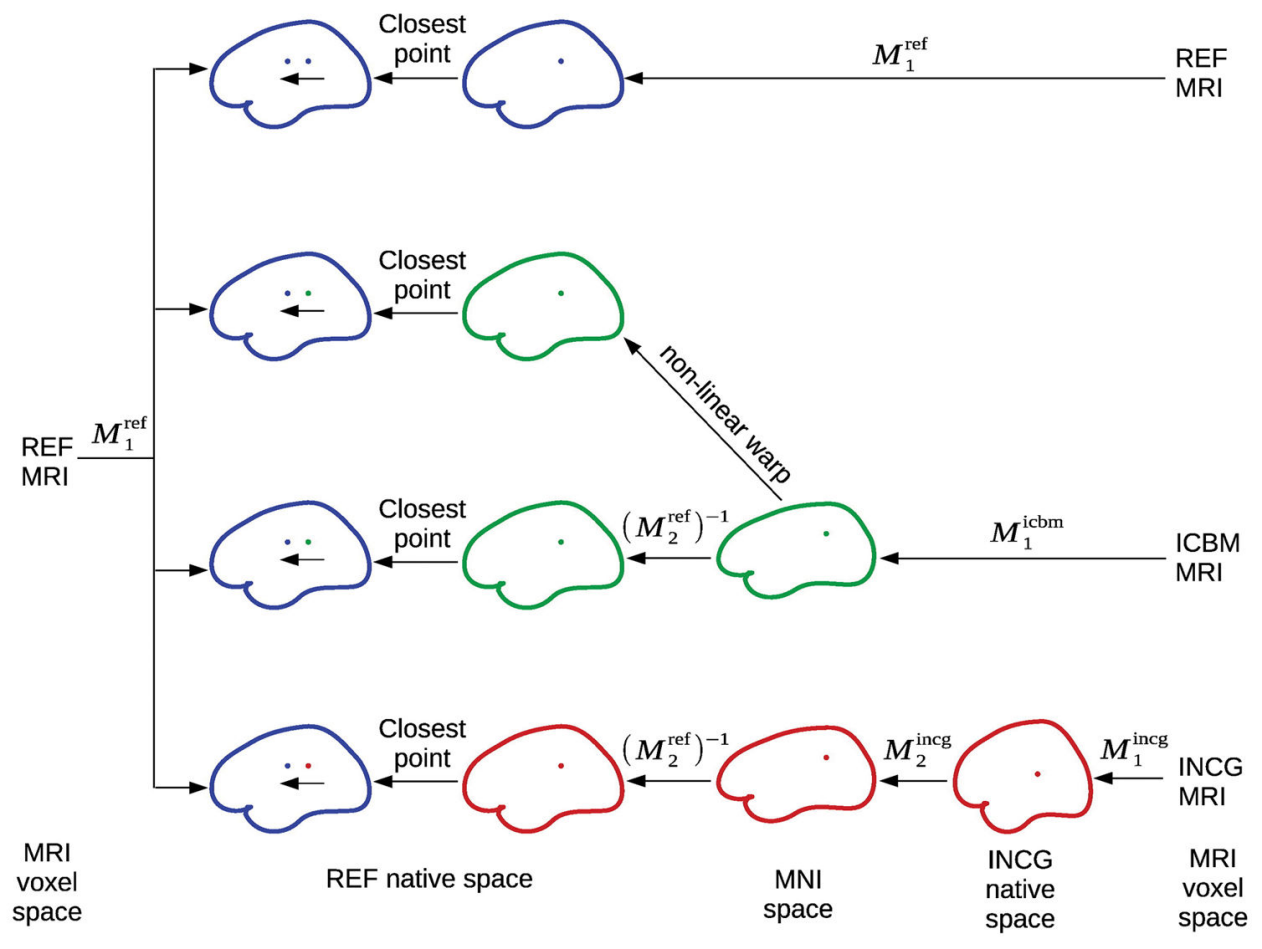
Mapping between different anatomies. *M*_1_ is a 6-parameter affine transform mapping locations from MRI voxel space into the native world-space as described in MRI acquisition and preprocessing section. *M*_2_ is a 12-parameter affine transform mapping locations from native world-space into MNI space. To identify matching points in the native space of the reference model REF FEM (blue), all locations are mapped into this space, and closest points in the two models are selected based on smallest Euclidean distance. REF BEM and REF SHE (blue, first row) are already in the native space of REF FEM. WARP BEM (green, second row) is in the correct space after being warped. ICBM BEM and ICBM-NY (green, third row) are mapped from the MNI space into the native space of the reference. INCG FEM (red, fourth row) is first mapped into MNI space and then mapped into the native space of the reference model. Data are never resampled in any of these mappings.

Comparisons between reference and approximate head models were carried out on 10,004 points covering the entire cortical surface for each head. To this end, mappings between locations in the reference anatomy and locations in the anatomy of the approximate head models had to be established (see Fig. 3). All anatomies were transformed into the native space of the reference head (blue). Models based on the reference anatomy (REF BEM and REF SHE; blue, top row in the figure) are already in that space and require no transformation. For WARP BEM (green, second row), the underlying ICBM152 anatomy is by construction aligned with the native space of the reference anatomy through the non-linear warping procedure applied within Brainstorm. For ICBM-NY and ICBM BEM (green, third row), the ICBM head was mapped from its native space (the MNI space) into the reference head’s native space using the inverse of the affine transformation $M_{2}^{\text{ref}}$ described in MRI acquisition and preprocessing section. For INCG FEM (red, fourth row), the same was achieved by consecutively applying the native-to-MNI affine transformation of the incongruent anatomy ( $M_{2}^{\text{incg}}$) and the MNI-to-native affine transformation of the reference anatomy (inverse of $M_{2}^{\text{ref}}$). Once model anatomies had been transformed to the reference head’s native space, matching locations were determined for each point of the reference model by selecting the closest point in the approximate anatomy in terms of Euclidean distance. Note that all spatial transformations were solely applied to the coordinates of the incongruent anatomies for the purpose of matching locations. The actual lead fields remained unchanged.

### Assessment of lead field approximation accuracy

We compared the lead fields of all approximate head models (ICBM-NY, REF BEM, REF SHE, INCG FEM, ICBM BEM, WARP BEM) to those of the reference model (REF FEM). To obtain topographical distributions of the errors, this comparison was performed separately for each location in the reference anatomy, where lead fields of the approximate head models were evaluated at the matching locations as outlined above.

Let the *M*×3 lead fields of the reference and incongruent model at the *i*th cortical location, **r***_i_*, be denoted by $L_{\text{ref}}^{i}$ and $L_{\text{appr}}^{i}$, where *M* is the number of electrodes. These lead fields are expressed with respect to the coordinate axes of the respective native spaces of the underlying anatomies, which are in general not aligned. Therefore, $L_{\text{ref}}^{i}$ and $L_{\text{appr}}^{i}$ are only comparable up to rotations. This problem could be circumvented by applying the spatial transformations between native spaces based on the transformation matrices *M*_2_ also to the lead fields. However, these transformations involve not only rotations but also scalings and shearings, which may bias the results. Instead of transforming the lead fields, we therefore decided to base our quantitative evaluation entirely on measures that are invariant to rotations in 3D space.

Adopting an EEG terminology, we compare lead fields in terms of the strength of their resulting scalp potentials relative to each other, as well as the similarity of these scalp potentials. The relative lead field strength (termed *gain*) at cortical location *i* is defined as.

(1) $$G_{i}=10\log_{10}\;\left(\frac{{\left\|L_{\text{ref}}^{i}\right\|}_{F}^{2}}{{\left\|L_{\text{appr}}^{i}\right\|}_{F}^{2}}\right),$$

and is measured on a dB scale. Here, ${\left\|\cdot \right\|}_{F}^{2}$ is the sum of the squared entries of a matrix. Note that *G_i_* is independent of the orientation of the source currents, as it is unaffected by arbitrary rotations $L_{\text{ref}}^{i}\leftarrow L_{\text{ref}}^{i}R_{t}$ and $L_{\text{appr}}^{i}\leftarrow L_{\text{appr}}^{i}R_{a}$ using orthogonal rotation matrices **R**_t_ and **R**_a_.

Lead field correlation is defined based on the largest principle angle between the subspaces spanned by $L_{\text{ref}}^{i}$ and $L_{\text{appr}}^{i}$ (Golub and Van Loan, 2012) and is computed using Matlab’s subspace command, again for each location *i*. Just as the gain, the subspace angle is independent of rotations within 3D space. However, while the gain measures exactly the scale ratio of two lead fields, the subspace angle is independent of any scaling. It is therefore a suitable measure of *subspace correlation*. Here we consider subspace angles normalized to the interval [0,1], where 1 stands for completely disjoint (orthogonal) lead fields, and 0 stands for lead fields that are identical up to arbitrary linear transformations. Subspace correlation is defined as 1 – subspace angle and is higher for more similar lead fields.

Notice that the two evaluation metrics were chosen because they approximately reflect the criteria used to measure tES targeting accuracy (gain), as well as to determine source locations in EEG source imaging (subspace correlation).

### Assessment of EEG source localization accuracy

We simulated an EEG inverse source reconstruction setting in order to assess the consequences of using an approximate head model in practical terms. Scalp potentials were generated for the reference head model REF FEM, while localization was carried out using either of the approximate models REF BEM, REF SHE, ICBM-NY, ICBM BEM, INCG FEM, and WARP BEM. Similar to the evaluation of approximation quality of the lead fields described above, the simulation was carried out separately for each cortical location, yielding a spatial distribution of localization errors. To this end, in the *i*-th run of the simulation, the lead field $L_{\text{ref}}^{i}$ at location **r***_i_* was projected onto the normal vector of the cortical surface at **r***_i_*, **n***_i_*, leading to a single *M*-dimensional vector $l_{\text{ref}}^{i}=L_{\text{ref}}^{i}n_{i}$ representing the scalp potential that would be generated by a dipolar current source at **r***_i_* oriented perpendicular to the cortical surface. This potential was subsequently regarded as a pseudo EEG measurement.

Localization was carried out by sweeping through all cortical locations **h***_j_* of the approximate head model and comparing $l_{\text{ref}}^{i}$ to $L_{\text{appr}}^{j}$ using the subspace correlation criterion. Note that this approach is similar to the classical ‘multiple signal classification’ (MUSIC) scan (Schmidt, 1986; Mosher and Leahy, 1999). The location **h**_*j*_*opt*__ leading to maximal subspace correlation was defined as the estimated source location in the approximate head model. After transforming **h**_*j*_*opt*__ to the native space of the reference head using the procedures outlined in Mapping between cortical locations of different anatomies section, the Euclidean distance to **r***_i_* was computed and defined the localization error. Note that, through the use of subspace correlation for defining source locations, this part of the evaluation is also invariant w.r.t. rotations of the native spaces of the reference and approximate anatomies.

### Assessment of tES targeting accuracy

The performance of the ICBM-NY was also evaluated in terms of tES targeting accuracy. In targeting of transcranial currents, models are used to optimize the current applied to each electrode location with the goal of increasing either focality or intensity of the stimulation in the brain (Im et al., 2008; Park et al., 2011; Dmochowski et al., 2011, 2013). Here we use the algorithm described in Dmochowski et al. (2011). Specifically, the electric field perpendicular to the cortical surface at the target location is maximized, with the total injected current being constrained to a safe limit (typically no more than 2 mA).

Each model may give different optimal electrode currents resulting in different electric field in the brain. To see how much the different models deviate from each other in this regard, we optimized intensity on target using the ‘ground truth’ model (REF FEM) as well as each of the approximate or incongruent models (REF BEM, REF SHE, ICBM-NY, ICBM BEM, INCG FEM, and WARP BEM). We did this for each cortical location **r***_i_* to obtain a corresponding optimal electric field distribution $E_{\text{ref}}^{i}$ (these are *N*×3 matrices with *N*=10,004 representing the number of cortical mesh points in the model). We also optimized intensity at the corresponding locations in the approximate models, where correspondence is determined following Mapping between cortical locations of different anatomies section,, and applied those optimal currents back to the reference model to obtain $E_{\text{appr}}^{i}$. This is the field distribution one would generate in the ‘true’ head if only approximate models were available for targeting.

Two metrics were defined to assess the targeting performance. The first one evaluates how different the intensities of the two electric fields $E_{\text{ref}}^{i}$ and $E_{\text{appr}}^{i}$ are at the target, measured by the relative

(2) $${\text{relErr}}^{i}=\frac{|E_{\text{appr}}^{i}(r_{i})-E_{\text{ref}}^{i}(r_{i})|}{E_{\text{ref}}^{i}(r_{i})}.$$

Here, *E^i^*(**r***_i_*) is the magnitude of the electric field at the target **r***_i_* when optimizing for that same target location. The second measure evaluates how well the peak intensities of the two fields $E_{\text{ref}}^{i}$ and $E_{\text{appr}}^{i}$ overlap on the cortex. This measure was used because clinicians are particularly interested in the areas of peak activation (presumed to correspond to desired neurophysiological effects). The Jaccard index (Jaccard, 1901) was used to quantify the similarity of the spatial distributions of the peak areas. It is given by

(3) $${\text{Jacc}}^{i}=\frac{|P_{\text{ref}}^{i}\cap P_{\text{appr}}^{i}|}{|P_{\text{ref}}^{\prime }\cup P_{\text{appr}}^{i}|},$$

where $P_{\text{ref}}^{i}$ and $P_{\text{appr}}^{i}$ are the peak areas (binary masks) corresponding to field intensities $E_{\text{ref}}^{i}$ and $E_{\text{appr}}^{i}$ larger than the 75th percentile. A Jaccard index close to 1 indicates perfect overlap between the two areas, while an index of 0 indicates that the two areas are entirely disjoint. relErr*^i^* and Jacc*^i^* were calculated for all cortical locations **r***_i_* in REF FEM, yielding a spatial distribution of targeting errors. Note that although in the approximate head model, the electric field is maximized along the normal direction of the cortical surface at the target, the two error metrics do not assume any fixed orientation of the electric field in the reference head and are hence invariant w.r.t. rotations between the native spaces of the reference and approximate anatomies.

For boundary element and spherical harmonics modeling, different conductivity values are used compared to FE modeling (Finite element modeling, Boundary element and spherical harmonics modeling, and Generation of individualized warped ICBM templates sections). Therefore, the intensity achieved at the target can be biased when using a BEM, WARP BEM, or SHE model to approximate the reference model REF FEM. This bias was corrected by computing an optimal global scalar coefficient that minimizes (in least-square sense) the difference between the lead fields of REF FEM and any non-FEM head model. This way, reference and approximate lead fields were brought to a similar scale.

## Results

**Fig. 4:**
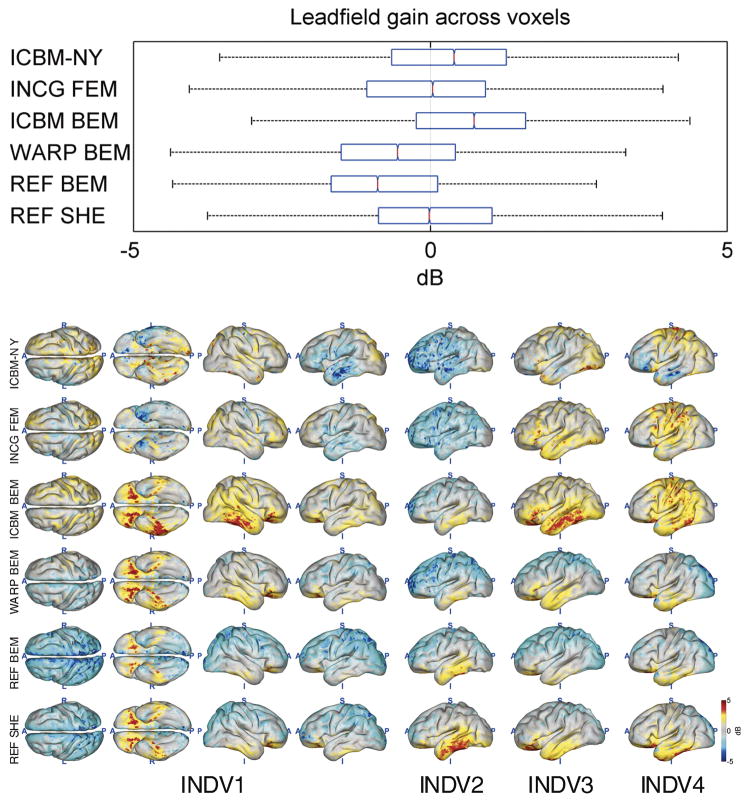
Lead field gain observed across all cortical locations when approximating a reference head model (a finite element model of an individual’s anatomy) by a head model that is either based on an incongruent anatomy or uses a different electrical model in the matching reference anatomy. Values closer to zero indicate better approximation performance. Approximation was carried out using ICBM-NY—the ‘New York Head’ model, INCG FEM—FEMs of three different individual anatomies incongruent with the one being tested, ICBM BEM—a boundary element model of the ICBM152 template, WARP BEM—a BEM of a version of the ICBM152 template that has been warped to fit the outer shape of the reference head, REF BEM—a BEM of the reference anatomy, and REF SHE—a spherical harmonics expansions model of the reference anatomy. Note that the lower three head models use individual information that is often not available in practice, and thus have an advantage over a fixed incongruent head. Upper panels: Median, 25th and 75th percentile, and most extreme values attained across the cortical locations of four individual subjects INDV1–4. Outliers are not plotted. Lower panels: topographical distributions of the gain for subject INDV1 (four views) and subjects INDV2–4 (left lateral view).

**Fig. 5:**
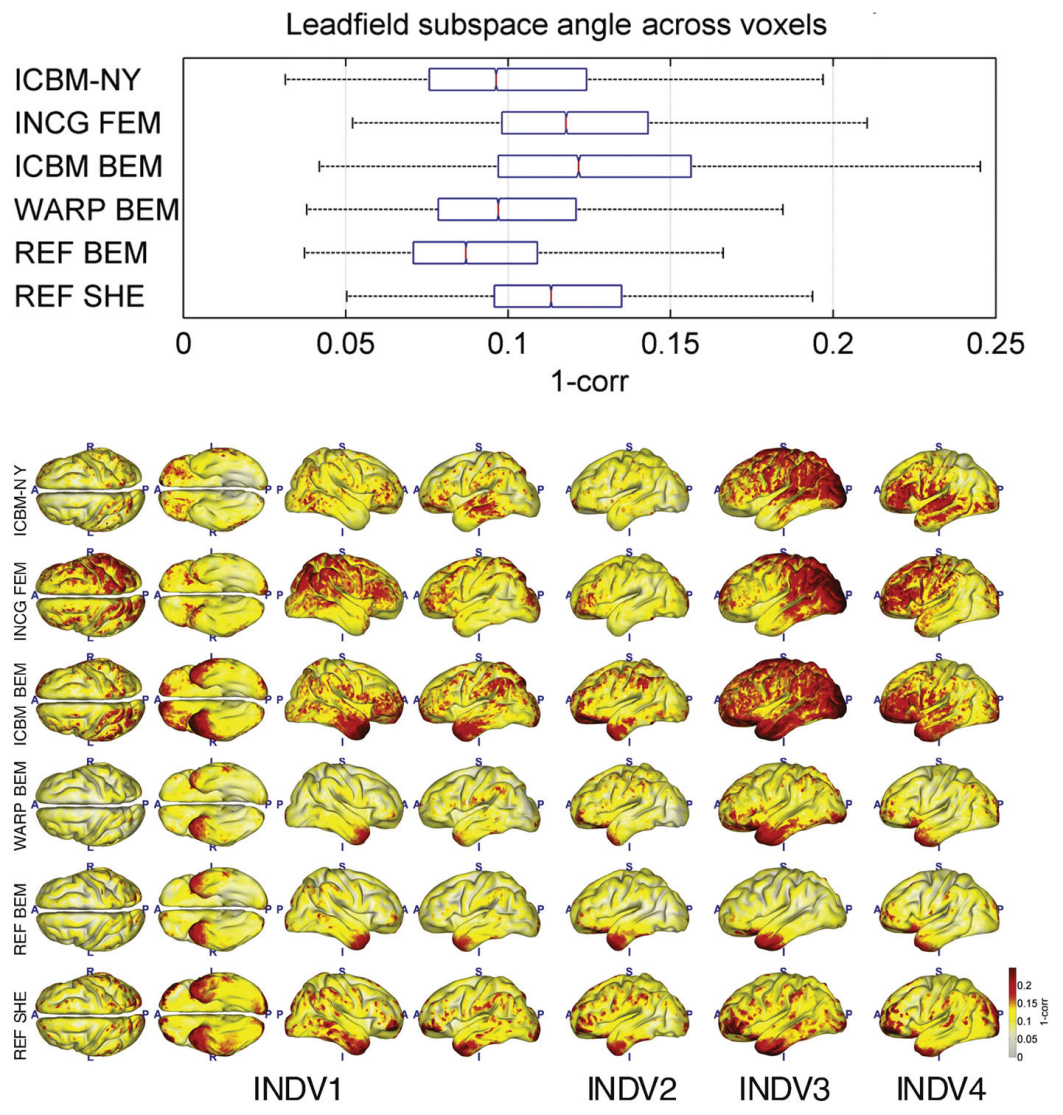
Subspace angle (1-subspace correlation) achieved across all cortical locations when approximating a reference head model (the FEM of an individual’s anatomy) by a head model that is either based on an incongruent anatomy or uses a different electrical model in the matching reference anatomy. Smaller values indicate better approximation performance. All graphs analogous to Fig. 4; see caption for detail.

**Fig. 6:**
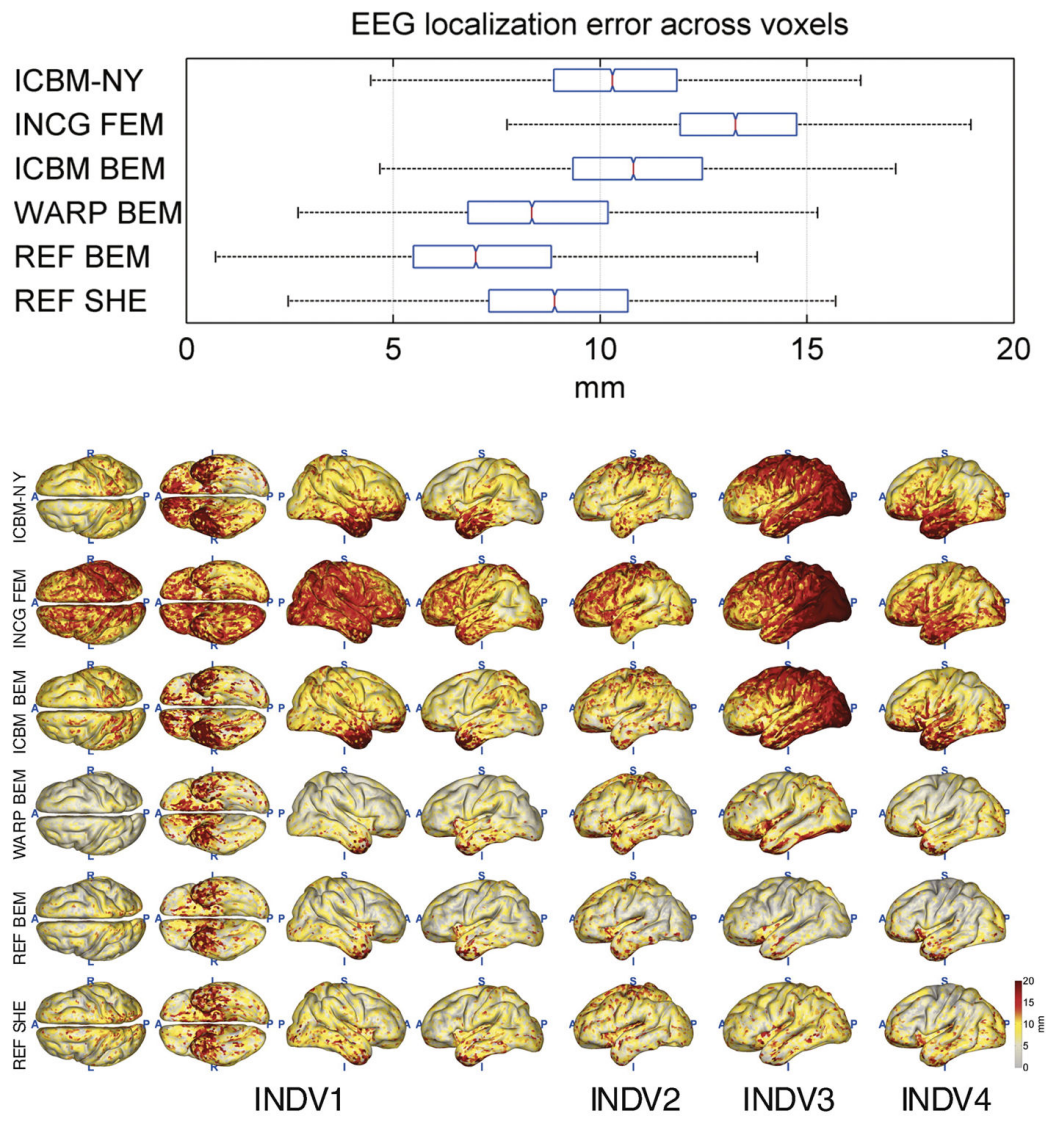
Localization error incurred for dipolar sources placed across all cortical locations when performing EEG source imaging in an approximate head model, which is either based on an incongruent anatomy or uses a different electrical model in the matching reference anatomy. All graphs analogous to Fig. 4; see caption for detail.

**Fig. 7:**
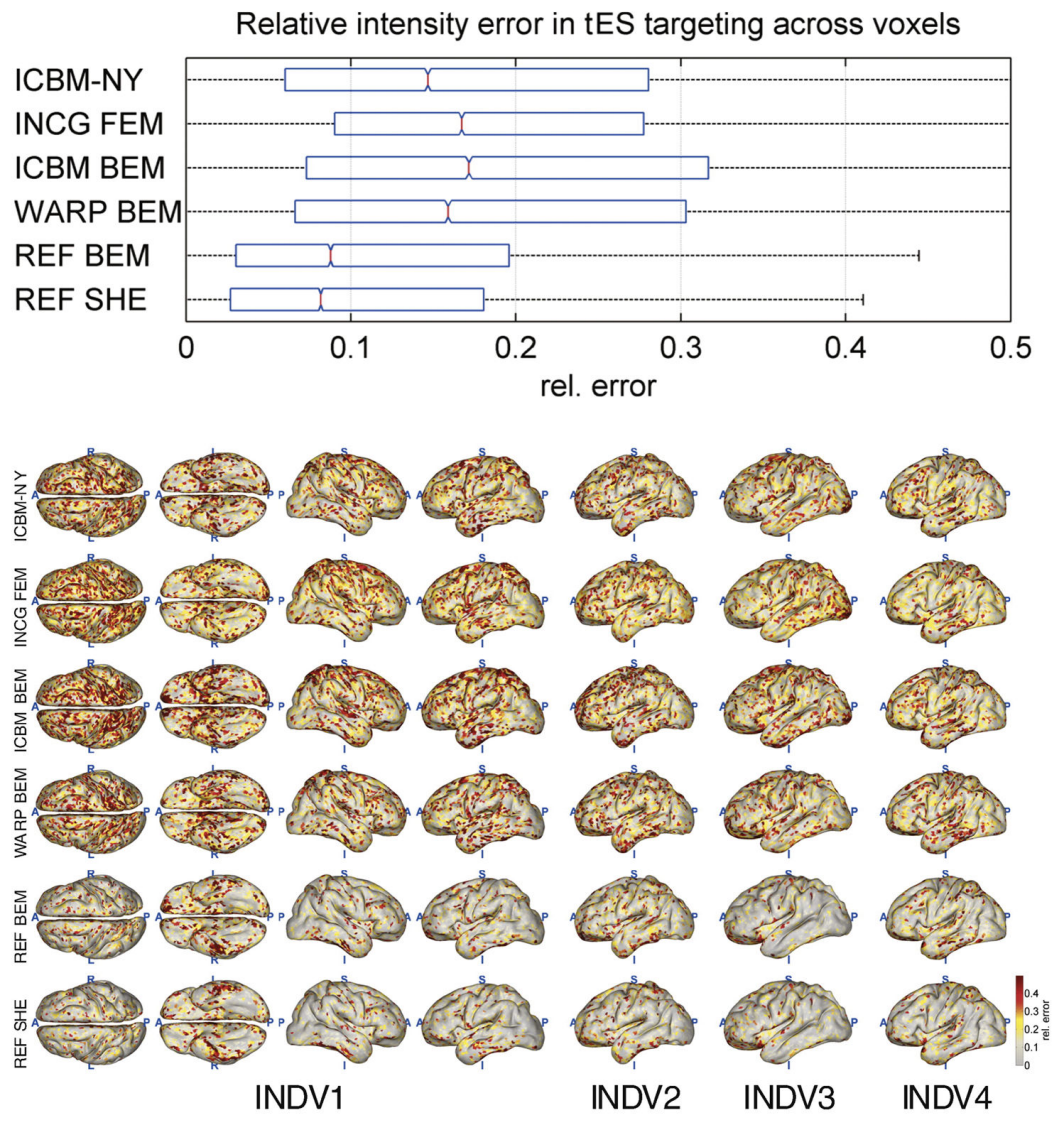
Relative error in electric field intensity incurred across all cortical locations when targeting a cortical location in individual subjects using an electrode montage optimized in a head model that is either based on an incongruent anatomy or uses a different electrical model in the matching reference anatomy. Smaller values indicate better targeting performance. All graphs analogous to Fig. 4; see caption for detail.

**Fig. 8:**
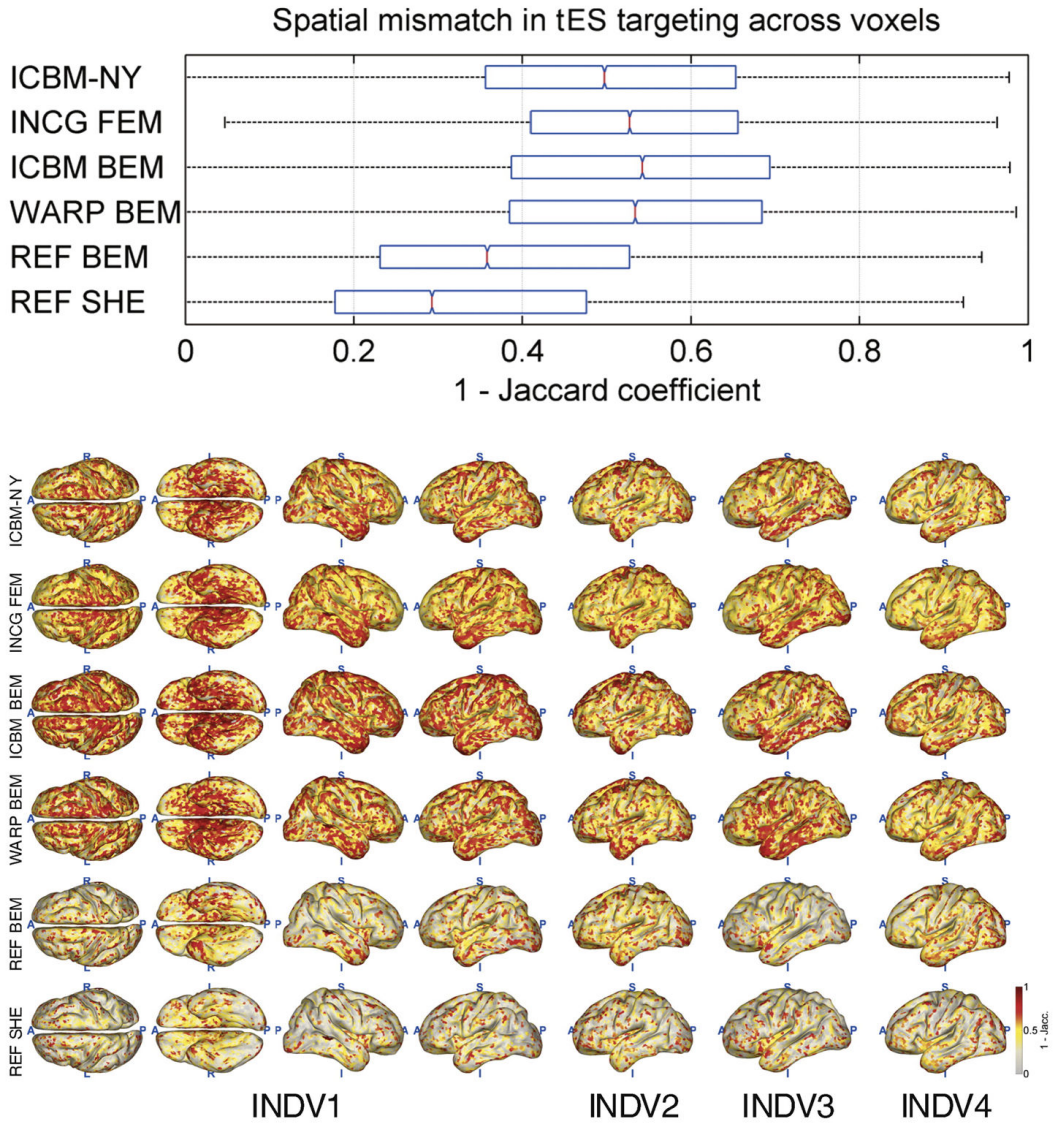
Jaccard spatial similarity index achieved across all cortical locations when targeting a cortical location. Smaller values indicate better targeting performance. All graphs analogous to Fig. 4; see caption for detail.

Figs. 4–8 depict the results of the quantitative evaluation of the ICBM-NY head model as compared to competing models in terms of five different error measures. The distributions of the errors shown in the upper panel of each figure are pooled over the four individual reference heads. In all instances, it is assumed that the individual FEMs (REF FEM) are the ‘ground truth,’ while the various other models are approximations. ICBM-NY provides the performance of the proposed New York Head when tested on the four individual FEMs. INCG FEM tests how well a detailed FEM of an individual can replicate another (incongruent) individual. Here, results are further pooled over the three incongruent individual heads serving as approximations (e.g., INDV2–4 when INDV1 is the reference anatomy). WARP BEM, REF BEM, and REF SHE indicate the results for various approximate BEM models tested on the four individual FEMs. The lower panels of the figures depict topographical distributions of the errors made for each of the four reference anatomies INDV1–4.

### Lead field approximation accuracy

Figs. 4 and 5 depict the results of the lead field approximation assessment in terms of gain and subspace correlation. The proposed ICBM-NY model as well as ICBM BEM slightly underestimates the global current intensity as compared to REF FEM. In contrast, REF BEM and WARP BEM slightly overestimate the overall current flow. REF SHE and the three incongruent individual models (INCG FEM) pooled together provide the most unbiased estimate of current flow. The range of gain factors attained by all models is relatively narrow, extending from −4 to 4 dB. In terms of subspace correlation (Fig. 5), ICBM-NY outperforms INCG FEM, ICBM BEM, and REF SHE, while being on par with WARP BEM. Here, ICBM-NY is only outperformed by a BEM computed in the reference anatomy (REF BEM).

The spatial distributions of the lead field approximation errors largely reflect the anatomical variation in our sample of four individual reference anatomies. A common pattern is, however, that models based on three-shell approximations (ICBM BEM, WARP BEM, REF BEM, and REF SHE) tend to overestimate the lead field intensity in more superficial frontal, central, parietal, and occipital regions and to underestimate the intensity in the deeper parts of the temporal lobe (lower panel of Fig. 4). FEMs (ICBM-NY and INCG FEM), in contrast, seem to overestimate the intensity in the temporal lobes. The subspace correlation (lower panel of Fig. 5) tends to be lowest in deep areas such as the tips of the temporal lobes for three-shell models, whereas for FEMs, the lowest correlations are achieved in frontal, parietal, or occipital areas depending on subject. Notably, the achieved subspace correlation differs substantially between subjects for ICBM-NY, INCG FEM, and ICBM BEM, whereas the variation for individual and individualized models (WARP BEM, REF BEM, and REF SHE) is much smaller.

### EEG source localization accuracy

Fig. 6 depicts the results of the EEG source localization study. ICBM-NY achieves a median localization error of 10.3 mm, outperforming INCG FEM (13.3 mm) and ICBM BEM (10.8 mm). However, individual and individualized models employing knowledge of the reference anatomy yield better localization performance (REF BEM: 6.9 mm, REF SHE: 8.9 mm, WARP BEM: 8.4 mm). The topographical distributions of the localization errors largely resemble the distributions of the lead field subspace correlations shown in Fig. 5, reflecting the choice of subspace correlation as the criterion for selecting source locations.

### tES targeting accuracy

Figs. 7 and 8 show the results of the tES targeting experiment. ICBM-NY outperforms INCG FEM, ICBM BEM, and even WARP BEM in terms of both the relative error of the achieved electric field intensity at the target and the Jaccard index of peak area distribution similarity. However, similar to what is observed in EEG source localization, ICBM-NY performs less well than REF BEM and REF SHE, as the latter models benefit from knowledge of the reference anatomy, which would require costly MR imaging in practice.

## Discussion

With the New York Head (ICBM-NY), we intended to create the most accurate general-purpose electrical volume conductor model possible today by integrating the currently most detailed anatomical templates of the average adult human head with state-of-the-art electrical and computational modeling. Our results indicate that the ICBM-NY is indeed highly competitive in terms of EEG source imaging and tES targeting. According to the performance metrics we evaluated, it outperforms arbitrary reference head models, as well as the relatively widely used BEM of the ICBM152. This suggests that one should use the New York Head for targeting and source localization whenever neither individual MR images nor digitized electrode coordinates are available. To facilitate using our model, all required data are made available online in Matlab format.

### Relation to the state-of-the-art

There are few software packages in the neuroimaging and neuromodulation communities to date that integrate the ICBM152 anatomical template as the reference model. The most commonly used ‘standard’ head is the Colin27 head (Holmes et al., 1998), included as a BEM in LORETA (Pascual-Marqui et al., 1994), EEGLAB-NFT (Acar and Makeig, 2010), Brainstorm (Tadel et al., 2011), and FieldTrip (Oostenveld et al., 2011); and as an FEM in COMETS (Jung et al., 2013) and BrainStimulator released with SCIRun 5.0 (Institute, 2015). Brainstorm added the ICBM152 v2009 (at 1 mm^3^ resolution) recently for boundary element modeling, but similar to Colin27, its FOV is limited to the brain area. The ICBM-NY, in contrast, employs highly detailed finite element modeling of six tissues including the CSF at 0.5 mm^3^ resolution. Its FOV moreover covers the entire head. This extended FOV is important for tES targeting, where it is common to place reference electrodes far away from the scalp (Huang et al., 2013).

An alternative to the ICBM-NY is a BEM of the ICBM152 head that is warped to an individual’s outer head shape (Leahy et al., 1998; Darvas et al., 2006; Acar and Makeig, 2010). This procedure can be used even in the absence of an individual MRI; however, it requires a (potentially error prone) digitization of the individual electrode positions. Our evaluation shows that warped ICBM152 models compare favorably against the ICBM-NY in terms of EEG source localization accuracy, but are outperformed by ICBM-NY with respect to tES targeting accuracy.

Another approach designed to replace individual head models in EEG source imaging has been proposed by (Valdés-Hernández et al., 2009). They used BEM to compute the lead fields for 305 individual heads, and then averaged either the cortical surfaces or the calculated lead fields in contrast to averaging the entire anatomy (MR images) of the head as was done to obtain ICBM152 and ICBM-NY. Their approach has been found to be more accurate than approaches based on averaged anatomies in terms of EEG source localization. However, for their study, the individual BEM was regarded as the ‘ground truth’ model. Furthermore, no assessment of tES targeting accuracy is provided.

### Limitations

The current evaluation is based on individual models of the heads of four Caucasian males serving as the ‘ground truth.’ Whether the ICBM-NY is a good approximation for the general population must be studied using larger numbers of more diverse reference heads. It also needs to be pointed out that the applicability of our model depends on the demographics of the population forming the original ICBM152 template. While an age range of 18.5–43.5 years has been reported in (Fonov et al., 2011), we are not aware of any additional demographic details in the literature describing the ICBM152 (Mazziotta et al., 1995, 2001a, 2001b; Grabner et al., 2006; Fonov et al., 2009, 2011).

One of our main goals here was to evaluate the ICBM-NY in terms practically relevant to the neuroimaging and neuromodulation communities; that is, in terms of EEG source localization and tES targeting performance. While the achieved accuracies arguably fall in ranges acceptable for most practical purposes (e.g., 10.3 mm average EEG source localization error), it should be noted that the results reported here comprise only those parts of the overall error that are due to approximate forward modeling. In practice, additional factors can substantially increase the overall error. Sources of error include incorrect electrode placement as well as high impedances due to insufficient contact between scalp and electrodes. In case of EEG, (measurement and physiological) noise represents an additional nuisance factor, as well as the fact that the EEG inverse problem is ill-posed and does typically not yield a unique solution. We minimized the influence of the latter two factors here by simulating only one cortical source at a time and by disregarding potential noise sources, enabling an unbiased comparison of head models. The variability of electrical conductivities across different individuals also contributes to the overall error. Lastly, it should be noted that even the ‘ground truth’ model of the reference head (in our case an FEM) is by definition only an approximation to the real world and contributes a share to the global error.

Point-like electrodes (see ##Segmentation and electrode placement section) are not entirely realistic in the context of tES, where sponge pads or high-definition disc electrodes are typically used (Nitsche and Paulus, 2000; Edwards et al., 2013). However, we did not perform realistic electrode modeling here, as our goal was to provide maximal flexibility w.r.t. electrode montage in order to make the ICBM-NY as widely applicable as possible. Modeling each electrode as a point allowed us to compute a single lead field for 231 candidate electrode locations covering the entire scalp. By selecting appropriate parts, that same lead field can be used for all montages involving subsets of these 231 electrode locations. Modeling a disc electrode with conductive gel underneath each of the 231 candidate locations would artificially increase the conductance of the scalp surface, and introduce errors for montages involving fewer than 231 electrodes, which is the default case in tES and even EEG. As an alternative, one might physically model specific electrode montages. However, in order to make such an approach widely applicable, this would have to be performed separately for each possible electrode montage, which is computationally prohibitive. An analysis of one bipolar montage (C4-Iz) shows that the electric field distribution in the brain obtained from using point-like electrodes only deviates by 4% from the field obtained using disc electrodes on average. It has also recently been shown that one can use an array of high-definition disc electrodes to approximate pad electrodes (Kempe et al., 2014).

Due to lack of diffusion tensor imaging (DTI) data for the ICBM152, we did not include WM anisotropy, nor did we differentiate between skull spongiosa and compacta for the ICBM-NY model. As a workaround, one could incorporate anisotropy by registering the diffusion tensor images of one arbitrary adult individual to the ICBM-NY anatomy. However, the result will be noisy because one individual cannot represent the average WM tractography across 152 subjects in the same way as the ICBM152 MRI does for the anatomy. Generally, it is still debatable whether or not WM anisotropy and inhomogeneous skull should be included in the modeling of EEG and tES. Many studies have shown that these two factors can lead to significant changes in the electric field distributions in the brain (Sadleir and Argibay, 2007; Dannhauer et al., 2011; Windhoff et al., 2011; Suh et al., 2012; Wagner et al., 2014). However, a recent study (Vorwerk et al., 2014) shows that explicit modeling of different skull layers might not be necessary especially when an optimized conductivity value is used, and it is admissible not to include white matter anisotropy considering the complexity and limitation of the modeling approach (e.g., uncertainties on converting diffusion imaging data into anisotropic conductivities (Shahid et al., 2013)). Most importantly, without validation from experimentally recorded data, no solid conclusion can be made regarding the necessity to model these details. Nevertheless, one should add this level of detail in the future when DTI data for ICBM152 and reliable modeling approaches become available.

### Evaluation criteria

The evaluation of tES targeting is sensitive to the orientation of the electric field at the target. The results presented here are based on maximizing the electric field along the normal direction of the cortical surface at the target. Further experiments show that, if the electric field is maximized without fixing its orientation at the target (i.e., maximizing its *magnitude*, (Dmochowski et al., 2013)), ICBM-NY performs better than all the BEMs (REF BEM, REF SHE, WARP BEM, ICBM BEM). The lack of the highly conductive CSF layer in 3-shell BEMs leads rather different current directions on the cortical surface as compared to the more realistic FEMs. There, shunting of currents by CSF results in predominant currents in direction normal to cortical surface. This systematic difference in field orientation introduces a bias if the electric field is maximized without considering its orientation at the target (BEM tends to have stronger fields in radial direction, whereas FEM tends to favour tangential fields). To avoid biases in the evaluation, we here optimized the field along the direction perpendicular to the cortical surface, which is the most physiologically meaningful orientation as the specific direction of the field is determined by the local anatomy of the cortex (i.e., radial at gyri and tangential at sulci). Analogously, we assumed normal oriented current when simulating source currents in the evaluation of EEG source imaging. It should be noted, however, that the error metrics used for tES targeting and EEG source imaging are invariant to field orientation, as they are computed using the field *magnitude* and *span*. Therefore, the variability of the lead fields due to differing native spaces does not affect the validity of the evaluation.

## Conclusions

We presented the New York Head (ICBM-NY), a highly detailed FEM of the average adult human head. The ICBM-NY integrates the currently most detailed anatomical templates with state-of-the-art electrical and computational modeling implementing the guidelines of (Vorwerk et al., 2014). Our model outperforms reference head models of ‘arbitrary’ individuals, as well as a BEM of the ICBM152 in terms of source localization and tES targeting accuracy. It is moreover competitive to individualized BEMs in terms of tES targeting accuracy. We therefore propose it as a new standard model for tES targeting and EEG source localization whenever an individual MRI is not available. All model data are made available online in Matlab format to facilitate broad adoption.

## Footnotes

1 Note that ICBM here only indicates that the head model is mainly derived from the ICBM152 template. We are not affiliated with or part of the ICBM.

2 Available at http://www.bic.mni.mcgill.ca/ServicesAtlases/HomePage.
